# Maxillary Postsurgical Ciliated Cysts (PSCCs): A Series of Three Cases

**DOI:** 10.1155/2024/5584515

**Published:** 2024-05-17

**Authors:** Domenico Sfondrini, Fabio Pagella, Matteo Pellegrini, Martina Ghizzoni, Andrea Scribante, Chiara Tore, Stefano Marelli

**Affiliations:** ^1^Maxillo-Facial Surgery Unit, Fondazione IRCCS Policlinico San Matteo, 27100 Pavia, Italy; ^2^Otorhinolaryngology Unit, Fondazione IRCCS Policlinico San Matteo, 27100 Pavia, Italy; ^3^Maxillofacial Surgery and Dental Unit, Fondazione IRCCS Cà Granda Ospedale Maggiore Policlinico, 20122 Milan, Italy; ^4^Department of Biomedical, Surgical and Dental Sciences, University of Milan, Via della Commenda 10, 20122 Milan, Italy; ^5^Section of Dentistry, Department of Clinical, Surgical, Diagnostic and Pediatric Sciences, University of Pavia, 27100 Pavia, Italy

## Abstract

**Introduction:**

A postsurgical ciliated cyst (PSCC) is an epithelial cyst that usually develops in the maxilla, although in rare cases, it can affect the mandible or other facial bones. The typical age of diagnosis is 40-50 years, with no gender prevalence, and the mean cyst development occurs approximately 10-15 years following a surgical or traumatic event. Some epithelial respiratory cells can be trapped into the bone tissue during maxillary surgical procedures or maxillary fractures. The pathogenetic mechanism can be attributed to an inflammatory process that stimulates epithelial proliferation, leading to cyst expansion caused by osmotic pressure difference.

**Methods:**

This study presents case series involving three surgical ciliated cysts located in the left maxilla, affecting two female patients (aged 49 and 55 years) and one male patient (aged 39 years). In all three cases, symptoms such as pain or swelling were mild and not consistently present. Two cases showed cyst development 10 and 15 years after implant placement, while one case was not associated with any surgical or traumatic event. CT scan identified well-defined unilocular lesions in the maxillary bone in each patient.

**Results:**

Histopathological examination of the surgical specimens confirmed the suspected diagnosis of a PSCC of maxilla. The cystic walls consisted of fibrous connective tissue with chronic inflammatory infiltrate, lined exclusively by a thin layer of ciliated pseudostratified columnar epithelium. In the third patient, it was not possible to rule out an unusual radicular cyst.

**Conclusions:**

Although PSCCs are not commonly encountered in daily practice, clinicians should consider this possibility including it in the differential diagnosis of odontogenic jaw cysts and benign jaw tumors, particularly in patients who have undergone previous surgeries in the maxillary area.

## 1. Introduction

A postsurgical ciliated cyst (PSCC), also known as respiratory implantation cyst, is an epithelial lesion that primarily develops in the maxilla following traumatic events or surgical procedures involving the maxillary sinus [1]. It can also rarely affect the mandible, typically occurring after double-jaw orthognathic surgery [2], and can potentially occur all over the facial bones. The pathogenetic mechanism can be attributed to the entrapment of some Schneiderian membrane cells (that cover the inner part of the maxillary sinus cavity [3]) or nasal mucosa cells into the bone tissue as a consequence of trauma or surgical procedures involving maxillary sinus or nasal mucosa. Orthognathic maxillary surgery, sinus lift augmentation, maxillary implant placement, and Caldwell-Luc antrostomy are the most often recognized causes [4–6].

Although very rare, upper molar tooth extraction or their retrograde root treatment has been reported in the PSCC pathogenesis [7].

These entrapped cells are probably triggered by an inflammatory process and proliferate to form a separate epithelium-lined cavity in which mucin is secreted with progressive enlargement [8]. This type of neoformation has a slow growth pattern with a mean development time of 18 years since the surgical or traumatic event [9], and the typical age of diagnosis is 40-50 years old, with no gender prevalence. It is usually incidentally discovered during routine radiological examinations, being often asymptomatic. If left undiagnosed or untreated, in cases of infection, pain can arise in the corresponding area, even with mucosal fistula development [10].

Other reported symptoms include nonspecific local swelling and discomfort frequently associated with a healthy mucosa [11, 12]. Radiologically, the cyst appears as a round, radiolucent, unilocular lesion with defined borders, similar to other jaw cysts [9, 13–16]. The purpose of this case series is to describe three patients who developed a PSCC in the upper left maxilla.

## 2. Case Series

### 2.1. Diagnosis and Etiology

Between 2022 and 2023, all three patients were referred to the Unit of Maxillo-Facial Surgery at IRCCS Policlinico San Matteo in Pavia, Italy.

#### 2.1.1. Case 1

**Figure 1:**
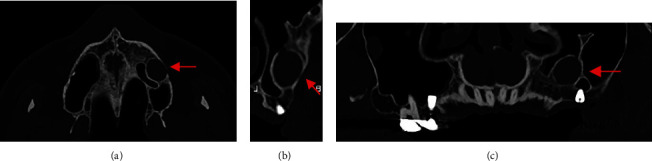
Case 1: preoperative CT scan showing round, unilocular, hypodense lesion (red arrows). (a) Axial view, (b) cross-sectional view, and (c) panoramic reconstruction view.

**Figure 2:**
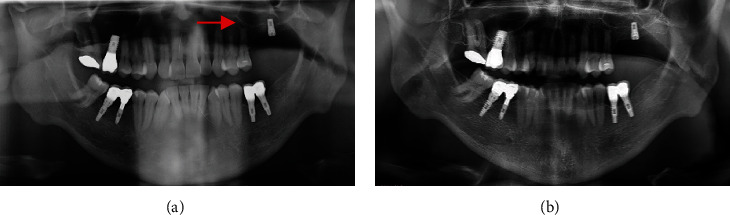
Case 1: (a) preoperative orthopantomography showing round, unilocular, radiolucent lesion developed in the region of lost dental implant (red arrow) and (b) postoperative orthopantomography.

A 39-year-old male patient underwent placement of two dental implants in the left maxillary molar region 10 years before presentation. The mesial one was lost after a few months, and the other one was left unloaded. He presented with swelling and pain in the corresponding area. An orthopantomography showed a radiolucent cystic lesion in the left maxillary bone. A CT was required for better surgical planning (Figures 1 and 2).

#### 2.1.2. Case 2

**Figure 3:**
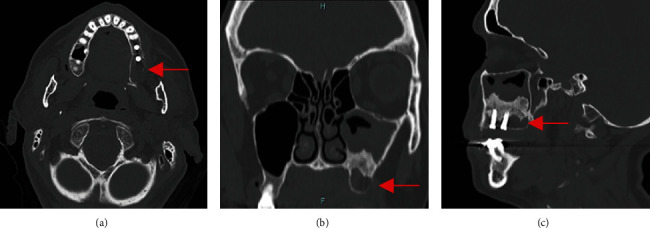
Case 2: (a) axial view preoperative CT scan showing hypodense lesion distal to posterior dental implant (red arrow) and (b, c) coronal and sagittal view showing the lesion (red arrow) developed under sinus lift xenograft.

A 49-year-old female patient reported a sinus lift procedure along with two implant placements in the upper left maxilla 15 years before consulting. The fixtures were left unloaded for economical reasons. A routine orthopantomography revealed a radiolucent lesion of 2 cm involving the distal fixture. There were no signs of swelling during the intraoral and extraoral examination, and the patient reported no symptoms. A CT scan was performed for surgical planning (Figure 3).

#### 2.1.3. Case 3

**Figure 4:**
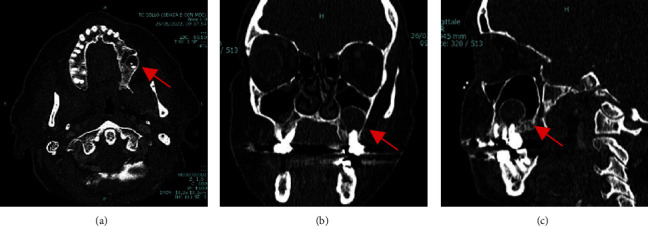
Case 3: preoperative CT scan showing round, hypodense lesion associated to the roots of a molar teeth, protruding in the left maxillary sinus (red arrows). (a) Axial view, (b) coronal view, and (c) sagittal view.

**Figure 5:**
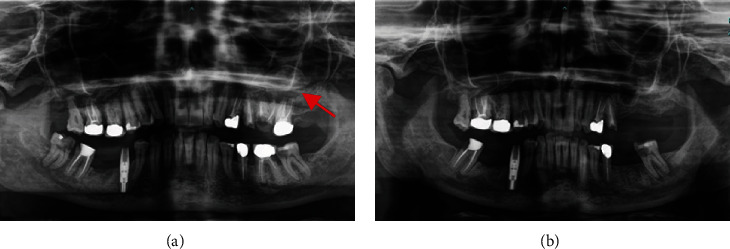
Case 3: (a) preoperative orthopantomography of case 3 showing radiolucent lesion in the left maxilla (red arrow) and (b) postoperative orthopantomography.

A 55-year-old female patient was referred for maxillary lesion incidentally found on orthopantomography as 2 cm round-shaped lesion with a thin radiopaque rim adjacent to the roots of left maxillary first and second molars. No previous history of maxillary trauma, surgeries, or sinusitis was recorded. The patient had previous endodontic treatment of the first and second left upper molars. There were no symptoms although intraoral inspection revealed a mild submucosal bulge in the upper left maxilla. A CT was performed to better determine the extent of the lesion and its relationships with adjacent anatomical structures (Figures 4 and 5).

### 2.2. Treatment Objectives

The treatment goal is the total removal of the cyst. In all three patients, the ciliated cyst was surgically removed (enucleation) with subsequent bone curettage of the residual cavity. In case of adjacent maxillary sinus membrane, special care was taken to preserve it.

### 2.3. Treatment Alternatives

The treatment approach for PSCC can vary based on factors such as cyst size, location, and anatomical relationships with adjacent structures. Treatment options can range from enucleation to marsupialization which is considered for larger cysts with significant cortical bone erosion and increased risk of jaw fractures [17, 18]. During marsupialization, the cyst wall is incised and sutured to the oral mucosa to create a pouch. This communication between the oral cavity and the cyst, reducing the intracystic pressure, leads to cyst volume decrease through peripheral bone neoformation. Once minimized the jaw's fracture risk, the cyst must be removed with enucleation. In this specific case series, marsupialization was not performed due to the small size of the cysts. Complete removal was possible without any risks of bone fractures or damage to adjacent anatomical structures. After surgical treatment, both clinical and radiological follow-ups are necessary to evaluate healing process and recurrences [19].

### 2.4. Treatment Progress

**Figure 6:**
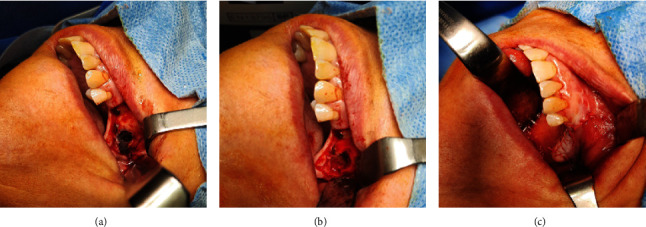
Intraoperative pictures of case 2: (a) mucosal flap raised and exposure of the cyst wall after ostectomy, (b) bone defect after cyst enucleation, and (c) suture after mucosal flap repositioning.

Complete cyst enucleation was performed under general anesthesia in all three cases. After local infiltration with anesthetic and vasoconstrictor (mepivacaine+adrenaline 1 : 100,000, Optocain, Molteni Dental Srl, Milan, Italy), a crestal incision with mesial and distal extensions was made using a no. 15 blade (Pic Solution, PikDare S.p.A, Como, Italy), and a mucoperiosteal flap was raised. When required, bone tissue was removed using a TPX round burr (Stryker, Kalamazoo, Michigan, USA), the cystic lining was exposed (Figure 6(a)), and the lesion was carefully removed (Figure 6(b)). The cysts appeared dark colored, because of the dark fluid content. All the specimens were sent to the Unit of Pathological Anatomy for the examination.

Following cyst enucleation, the surgical site was curetted and cleansed with saline solution. The mucoperiosteal flap was repositioned, and suture (4.0 VICRYL RAPIDE, Ethicon, Somerville, New Jersey, USA) was placed along the incision (Figure 6(c)).

### 2.5. Treatment Results

Following the surgical enucleation, all three cases experienced uneventful healing. During the monthly follow-up (minimum 12 months), none of the patients displayed signs of infection or recurrence.

Histopathological examination of the surgical specimens confirmed the suspected diagnosis of PSCC. The macroscopic dimensions of the collected samples varied from 1.2 × 1 × 0.7 to 1.8 × 0.3 × 0.3 cm.

**Figure 7:**
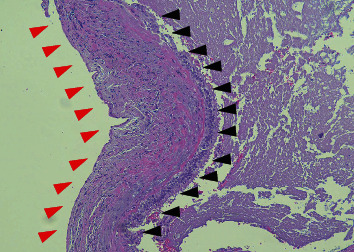
Haematoxylin and eosin-stained section showing the histopathologic features of a surgical ciliated cyst. The cyst wall consists of fibrous connective tissue wall (red arrows head) lined by a pseudostratified columnar ciliated epithelium (black arrow head) (original magnification 200x).

Microscopic examination revealed a benign cystic neoformation, characterized by a ciliated columnar epithelial lining, with no evidence of atypia, hemosiderin deposits, or calcification (Figure 7). Additionally, images indicating the presence of chronic inflammation were observed.

## 3. Discussion

Several cases of PSCC following surgeries have been reported in the literature.

Named in different ways, they are frequently reported as postoperative maxillary cysts because they have been firstly described as a complication of radical maxillary surgery for sinusitis treatment [20]. Although the maxillary bone is still the most affected site today, these lesions can potentially involve all the facial bones, following a nasomaxillary fracture or a surgical procedure. To date, they are generally referred to as postsurgical ciliated cyst (PSCC).

PSCC is a benign unilocular slow-growing lesion which can mimic other cysts and tumors of maxilla region, causing severe bone defects, cortical plate thinning, and facial aesthetic impairment especially if left untreated for a long time [21, 22].

PSCC typically presents as asymptomatic, despite symptoms like pain and swelling can occasionally manifest. The clinical behavior, combined with radiological imaging, does not lead often to a specific diagnosis compared to other jaw cysts. A positive history of nasomaxillary fracture or past surgical maxillary procedures can guide clinicians toward a suspected diagnosis, later confirmed by histopathological examination [23, 24].

Various surgical procedures ranging from traumatic dental extraction, apicoectomy, and orthognathic surgery to Caldwell-Luc antrostomy can lead to PSCC growth.

After surgery or facial bone fracture creating a wound in the maxillary antrum or nasal mucosa, respiratory epithelium can remain entrapped into the bone and leads to a cyst development. Furthermore, these cells can remain attached to the surgical instruments used to perform maxillary or nasal surgery and potentially transferred in other surgical sites, usually in the mandibular area [25, 26].

Ciliated cyst development in the mandible is exceptionally rare, with fewer than 20 cases reported to date [27]. Simultaneous surgical procedures involving both the maxilla and mandible, utilizing the same instruments, have been implicated in the mandibular PSCC development [28]. It can occur anteriorly in the chin region in patients who underwent bone genioplasty after LeFort I osteotomy, or more posteriorly, in the sagittal split osteotomy region, following bimaxillary orthognathic surgeries [29, 30].

Moreover, as reported in this study, PSCC can develop as a long-term subsequence after sinus lift surgery or maxillary dental implant placement [23, 31, 32]. Mucous membrane tearing, a common complication of this procedure, can lead to epithelial entrapment and cyst development [33, 34].

Although these cases are considered rare, the possibility of their occurrence cannot be ignored and should be included in the informed consent discussions.

As suggested by many authors [11], prevention of PSCC can be possible with meticulous surgical technique, including suturing ripped nasal mucosa, removing sinus mucosa during orthognathic or facial trauma procedures, cleaning or changing the saw blades after maxillary osteotomy, avoiding entrapped mucosa between maxillary bones, doing mandible surgery first, and meticulous maintenance of the Schneiderian membrane in sinus lift.

In the first patient, the PSCC developed 10 years after maxillary implant placement and affected only the cranial portion of the fixture. Being the occlusal bone around the implant preserved, the authors decided to leave it in place after cyst enucleation, as reported by other authors [35].

Case no. 3 deserves a separate discussion: the authors presented a PSCC in a patient without any previous surgery or trauma reported in maxillary bone or in the paranasal area. The first and second molar endodontic treatment was the only procedure referred, as seen in orthopantomography (Figure 5).

Suspecting a radicular cyst, the authors performed a surgical cyst removal together with the first and second molar teeth avulsion, previously endodontically treated. On the contrary, the histological evaluation reported ciliated pseudostratified columnar epithelium lining the cyst lumen like in PSCCs.

In the literature, endodontic surgery (apicoectomy) of the upper molar teeth has been widely described as a possible cause of PSCC development [36] while only one case of PSCC following orthograde root canal treatment has been reported [37].

The epithelium that usually lines the cavities of radicular cysts is classified as stratified squamous epithelium. In some cases, radicular cyst cavities can be partially or totally lined by a respiratory epithelium, the ciliated pseudostratified columnar epithelium [38–40]: this histological finding, even if extremely rare in odontogenic cysts, involves more often the superior upper molar region, in proximity to the sinus mucosa, although it has also been reported in some radicular mandibular cysts. This phenomenon, according to different authors, represents the 0.9%-8% of radicular cyst [36, 41, 42], and it is supposed to be the consequence of a special form of metaplasia, or more likely due to sinus cell involvement in the radicular cyst growth of the upper molar teeth [36].

Without a history of surgical trauma in the maxilla, an endodontic origin should be suspected. Consequently, a surgical endodontic treatment or avulsion of the involved teeth should be performed together with cyst removal.

In this case, even the histopathology cannot distinguish between a radicular cyst with respiratory features and a PSCC.

Potentially, the differential diagnosis of maxillary PSCC must be with all maxillary unilocular mass expanding into the maxillary sinus (antral lesions) or toward it, like the extra-antral lesions. The last one is represented by odontogenic tumor (e.g., ameloblastoma and odontogenic fibroma) and nonodontogenic benign tumor (e.g., central giant cell granuloma). However, these neoplasms typically exhibit other distinguishable radiological features [43].

The most frequent extra-antral lesions are the odontogenic and nonodontogenic maxillary cysts: these arise within the alveolar process and expand the sinus floor upward. Very often, a bone plate between cystic mass and maxillary antrum is observed on CT scan: this feature can identify clearly the extra-antral cyst from the true antral lesions, like mucocele.

Mucocele is a sac that is lined with respiratory epithelium and contains mucus. There are two types of mucoceles. Primary mucoceles are referred to as mucous retention cysts and are small, thin-walled cysts, generally found incidentally [44]. Secondary mucocele can develop in case of obstruction of sinus ostium caused by benign and malignant lesions, chronic mucosal infection, or allergic disease. This mucocele is an expansion of the maxillary antrum with mucous retention that grows eccentrically and involves the whole maxillary antrum, with localized bony wall erosion. Some authors consider the PSCC a special type of secondary mucocele [45, 46] and could be either antral or extra-antral growing lesion, depending on the cause.

Another entity to consider among the antral lesions is the pseudocysts, most commonly found incidentally in the maxillary sinus on the orthopantomography. Antral pseudocysts are simple inflammatory exudate between periosteum and antral mucosa, without epithelial lining, unlike the PSCC and other true cysts. No treatment is required. Radiologically, it is easy to recognize by its dome-shaped radiopacity on uninterrupted bony sinus floor.

This study does have limitations, being a case series with only a 12-month follow-up. A lack of a comparison group and potentially short follow-up duration may impact generalizability and validation.

## 4. Conclusion

While surgical ciliated cysts may not be commonly encountered in routine practice, clinicians should be vigilant and include them in the differential diagnosis of both odontogenic and nonodontogenic cysts of the jaws, particularly in patients with a history of prior surgeries in the maxillary area. However, even rarely, these cysts can also occur after tooth extraction or endodontic treatment of the upper molar or premolar (in continuity with the sinus floor mucosa). Further research is needed to validate these findings and improve understanding of PSCC.

## 5. Take Home Message

The development of postsurgical ciliated cysts should be considered in informed consent discussions for all the surgical procedures involving the sinus mucosa.

Prevention of adopting meticulous surgical techniques should be emphasized.

## Data Availability

The authors confirm that the data supporting the findings of this study are available within the article.
